# Supplementary material for: The draft genome of Cochliopodium minus reveals a complete meiosis toolkit and provides insight into the evolution of sexual mechanisms in Amoebozoa
Source: Sci Rep. 2022 Jun 14;12:9841. doi: 10.1038/s41598-022-14131-y (PMC9198077; doi:10.1038/s41598-022-14131-y)
Supplement: Supplementary file 1 — Supplementary Information 1. [file 41598_2022_14131_MOESM1_ESM.docx]

**Repetitive elements of *Cochliopodium minus* draft genome**

In order to identify repeat elements in the *C. minus* draft genome assembly, RepeatMasker (Omicsbox v2.0.36) was used with the *soft masking* option. Repeat masking was done using HMMER search engine along with the eukaryotic database. Our analysis revealed that the draft genome of *Cochliopodium minus* is composed of ~93.7% non-repetitive DNA (Appendix Fig. 1). The remaining ~6.3% are composed of various elements including short-interspersed nuclear elements (SINEs), Long interspersed nuclear elements (LINEs), long terminal repeats (LTRs), DNA transposons, Rolling-circles, small RNA, Satellites, simple repeats and low complexity regions (Appendix Fig. 1). The latter two make up most of the repetitive genomic region (6.31%). Repetitive genomic regions are among the hardest regions to assembly. Accurate predictions of these regions require high quality and long-reads. Given the complex nature of the repetitive regions, this preliminary result needs further analysis.

**Appendix Figure 1**. Genomic composition of the draft genome of *Cochliopodium minus*.

**Mitochondrial genome of *Cochliopodium minus***

Our assembly of mitochondrial genome resulted in a total size of 52,063 bps (Appendix Figs. 2, 3). The mitochondrial genome is composed of the complexes of electron transport chain (NADH, Cytochromes), ATP synthase, ribosomal-proteins and -RNAs as well as transfer RNAs (Appendix Figs. 2, 3). Our genomic data mostly comes from nuclear pellets and most of the mitochondria were removed along with bacteria contaminants. This step was necessary in order to minimize contamination from food bacteria. As a result, our attempt to reconstruct a circular mitochondrial genome of *Cochliopodium minus* likely indicates its incompleteness (Appendix Fig. 3). Nevertheless, our mitochondrial genome consisted of most of the well-known genes described in amoebae and other eukaryotes.

**Appendix Figure 2**. Genomic composition of *Cochliopodium minus* mitochondrial genome analyzed using MITOS (http://mitos.bioinf.uni-leipzig.de/index.py)^1^.

**Appendix Figure 3**. Circular mitochondrial genome of *Cochliopodium minus* showing gene arrangements reconstructed using OGDRAW (https://chlorobox.mpimp-golm.mpg.de/OGDraw.html%20)^2^. Refer Appendix Figure 2 for the complete list of genes.

1 Bernt, M. *et al.* MITOS: improved de novo metazoan mitochondrial genome annotation. *Mol Phylogenet Evol* **69**, 313-319, doi:10.1016/j.ympev.2012.08.023 (2013).

2 Greiner, S., Lehwark, P. & Bock, R. OrganellarGenomeDRAW (OGDRAW) version 1.3.1: expanded toolkit for the graphical visualization of organellar genomes. *Nucleic Acids Res* **47**, W59-W64, doi:10.1093/nar/gkz238 (2019).
