# Supplementary figures and images for: The draft genome of Cochliopodium minus reveals a complete meiosis toolkit and provides insight into the evolution of sexual mechanisms in Amoebozoa
Source: Sci Rep. 2022 Jun 14;12:9841. doi: 10.1038/s41598-022-14131-y (PMC9198077; doi:10.1038/s41598-022-14131-y)

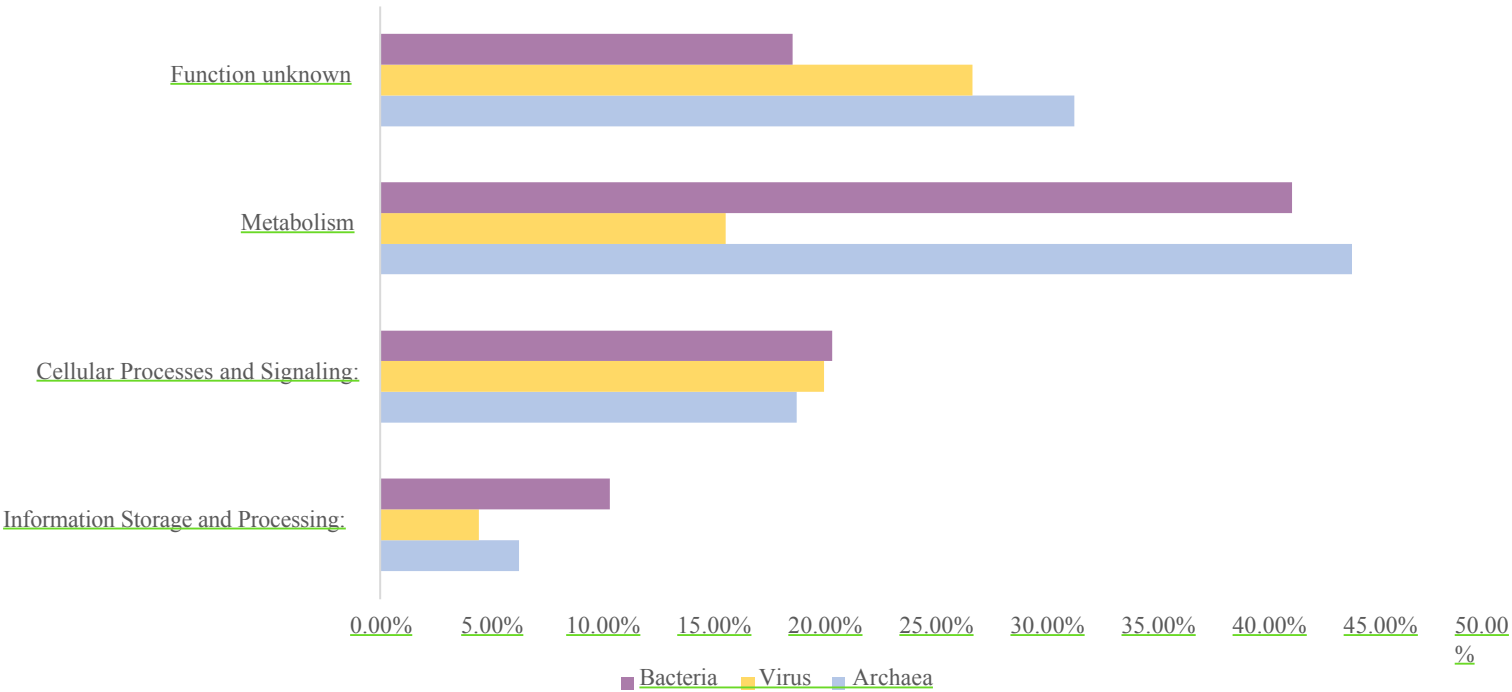

Supplement: Supplementary file 2 — Supplementary Figure S1. [file 41598_2022_14131_MOESM2_ESM.pdf]

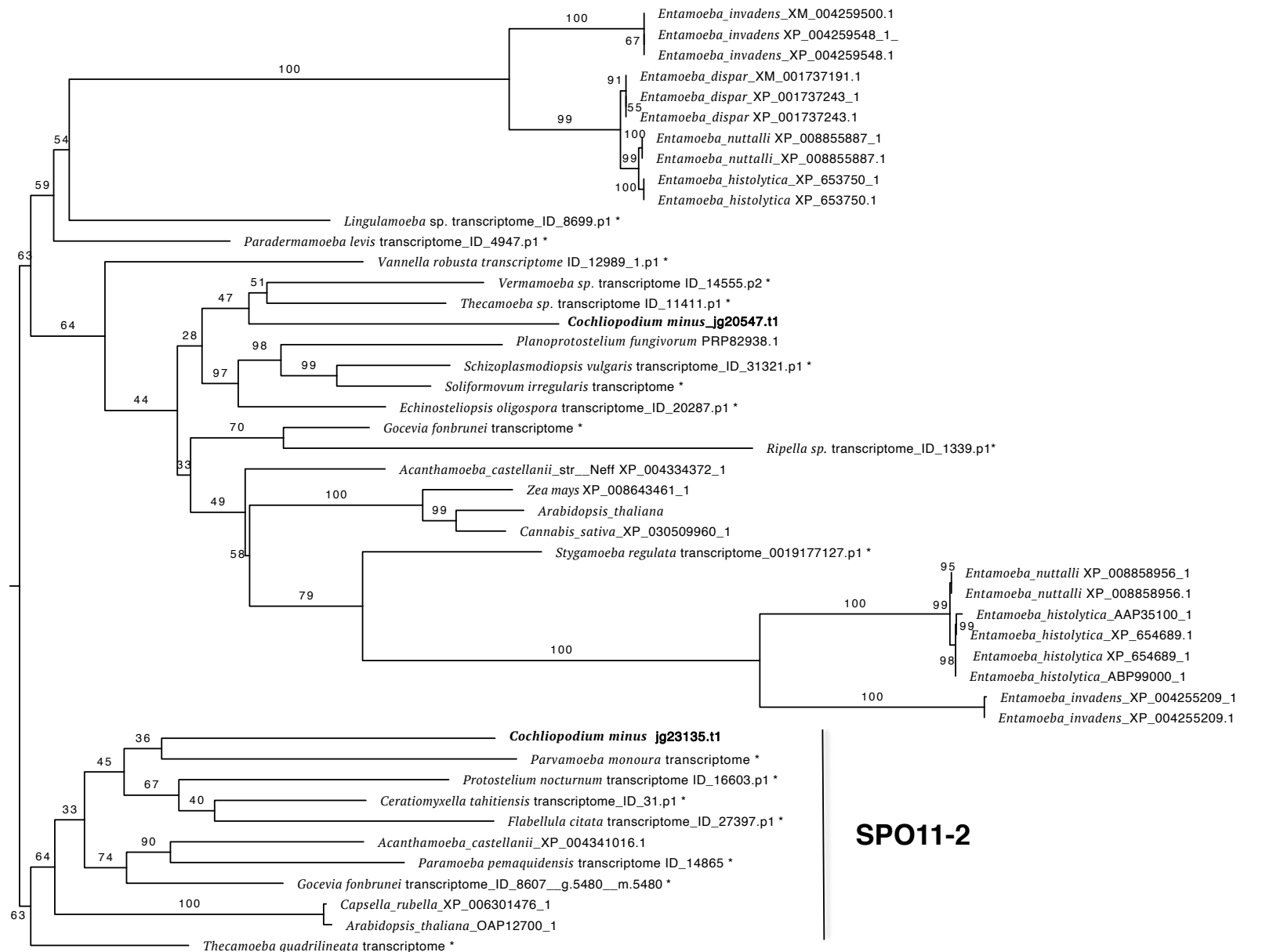

SPO11-1

SPO11-2

Supplement: Supplementary file 3 — Supplementary Figure S2. [file 41598_2022_14131_MOESM3_ESM.pdf]

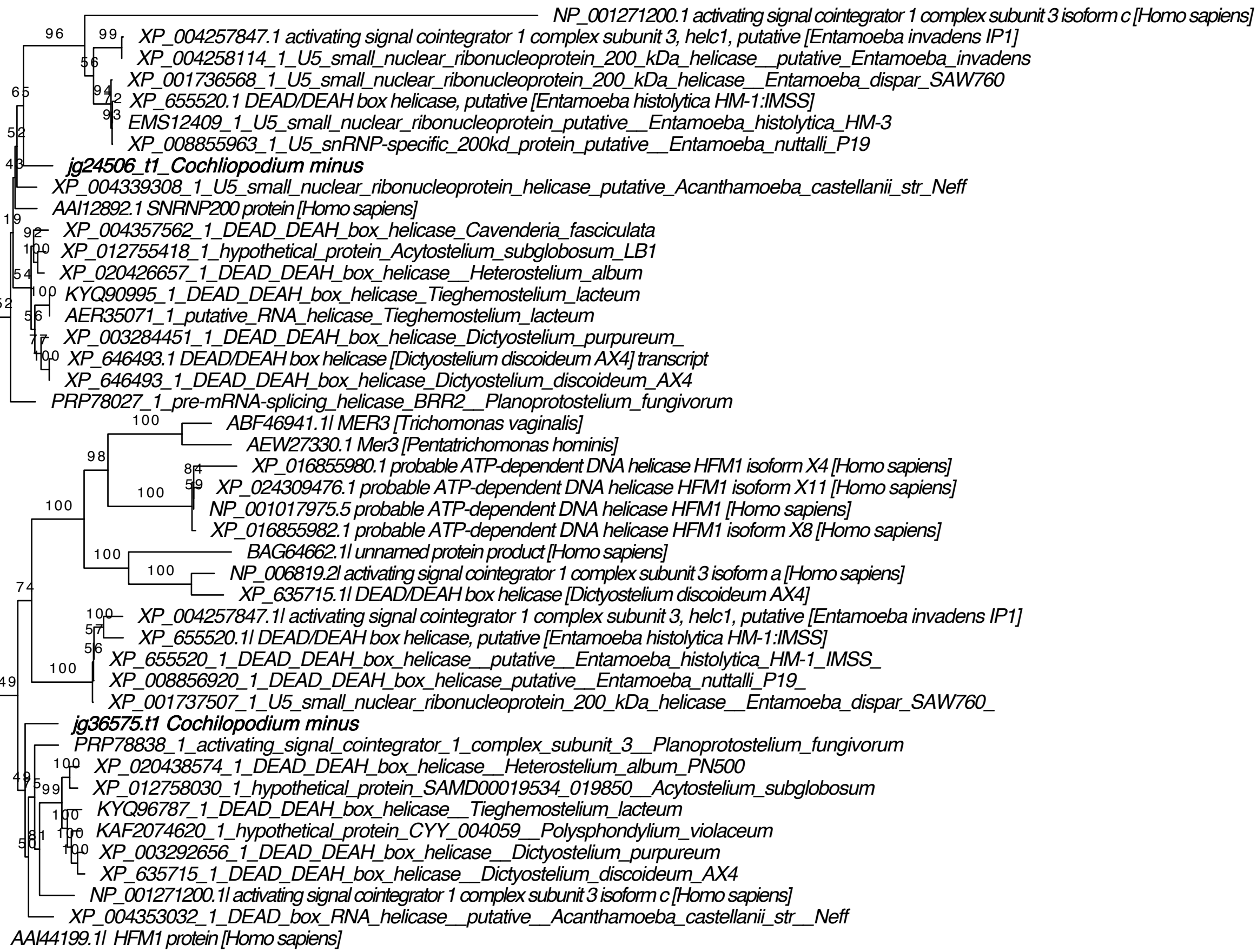

Supplement: Supplementary file 4 — Supplementary Figure S3. [file 41598_2022_14131_MOESM4_ESM.pdf]
