# Supplementary material for: The draft genome of Cochliopodium minus reveals a complete meiosis toolkit and provides insight into the evolution of sexual mechanisms in Amoebozoa
Source: Sci Rep. 2022 Jun 14;12:9841. doi: 10.1038/s41598-022-14131-y (PMC9198077; doi:10.1038/s41598-022-14131-y)
Supplement: Supplementary file 5 — Supplementary Legends. [file 41598_2022_14131_MOESM5_ESM.docx]

**Supplementary Materials Captions**

**Supplementary file 1.** *Cochilopodium minus* de novo Genome Assembly and Annotation from start to finish.

**Figure S1**. Functional categories based on Cluster Orthologous Groups (COGs) database of putative LGTs in bacteria, viruses and archaea.

**Figure S2**. Phylogenetic reconstructions of SPO11 paralogs including more amoebozoan taxa using transcriptome data. Transcript from some amoebae group with *SPO11-2* supporting the prevalence of *SPO11-2* in other amoebozoan lineages. Among amoebae with transcriptome data, *Gocevia fonbrunei* has both copies. Note the presence of in-paralog (lineage specific duplication) of *SPO11-1* in the parasitic genus *Entamoeba*. Clade supports at nodes are ML IQ-TREE 1000 ultrafast bootstrap values. All branches are drawn to scale.

**Figure S3**. Phylogenetic reconstructions of MER3 paralogs. Two copies of MER3 genes from the *Cochliopodium minus* draft genome are placed into two separate poorly supported clades. The copy, jg36575.t1, displaying alternative splicing and that is highly expressed in fused cells of *C. minus* fall with canonical copy of MER3 found in other eukaryotes. Clade supports at nodes are ML IQ-TREE 1000 ultrafast bootstrap values. All branches are drawn to scale.

**Table S1**. Distribution of the gene models of *Cochliopodium minus* draft genome in categories of clusters of orthologous groups of proteins (COGs).

**Table S2**. Differential Gene Expression of ORFan genes using PFAM scan between fused *versus* unfused cells of *Cochliopodium minus*.

**Table S3**. Putative LGT-derived genes in *Cochliopodium minus* genome with Alien Index above threshold (>45) scores.

**Table S4**. Virus matching gene models of *Cochliopodium minus* genome.

**Table S5**. Tyrosine Kinase genes-based on InterProScan analysis of the *Cochliopodium minus* genome.

**Table S6**. Genome size, gene content and GC content of amoebae that are annotated and unannotated genome available in the NCBI.
