# Supplementary material for: The draft genome of Cochliopodium minus reveals a complete meiosis toolkit and provides insight into the evolution of sexual mechanisms in Amoebozoa
Source: Sci Rep. 2022 Jun 14;12:9841. doi: 10.1038/s41598-022-14131-y (PMC9198077; doi:10.1038/s41598-022-14131-y)
Supplement: Supplementary file 6 — Supplementary Information 2. [file 41598_2022_14131_MOESM6_ESM.docx]

# *Cochilopodium minus* de novo Genome Assembly and Annotation from start to finish

**The Draft Genome of *Cochliopodium minus* reveals a complete meiosis toolkit and provides insight into the evolution of sexual mechanisms in Amoebozoa**

Yonas I. Tekle^*1^, Fang Wang^1£^, Hanh Tran^1£^, T. Danielle Hayes^2, 3^, Joseph F. Ryan^2,4^

^1^Dept. of Biology, Spelman College, Atlanta, Georgia, USA

^2^Whitney Laboratory for Marine Bioscience, University of Florida, St. Augustine, FL, USA

^3^Iowa State University, Ames, Iowa, USA

^4^Dept. of Biology, University of Florida, Gainesville, Florida, USA

^£^These authors contributed equally.

*Corresponding Author: Yonas I. Tekle, Spelman College, 350 Spelman Lane Southwest, Atlanta, GA 30314---Telephone number: 404-270-5779; e-mail: ytekle@spelman.edu

# PART 1: Genome Assembly

#### Trim adapters from Illumina paired end reads using BBDUK*

#### * https://jgi.doe.gov/data-and-tools/bbtools/bb-tools-user-guide/bbduk-guide/

## trimming adapters and low quality reads

bbduk.sh in1=/YT11_R1_001_trimq10.fastq.gz in2=//YT11_R2_001_trimq10.fastq.gz out1=YT11_R1_001_trimq25.fastq.gz out2=YT11_R2_001_trimq25.fastq.gz minlen=100 qtrim=rl trimq=25 hdist=1 stats=YT11_bbduk_trimq25_Stats.txt

#### 2. Assembly 10x Genomics reads with Supernova^1^

supernova run --id=N5_10xAssembly --fastqs=/pylon5/tr5fpup/ytekle/fwang/Genome_projects/N5_10x_genomics_data/Raw_reads --localmem=1000 --localcores=30 # Run the Supernova assembly process

#### 3. Assemble Nanopore data using Canu v2.2^2-4^

canu genomeSize=50m -p Cminus_contigs -nanopore-raw N5_ONT_YT11.fastq

#### 4. Assemble Illumina paired end reads with SPAdes v3.14.1^5^

spades.py -1 YT11_R1_001_trimq25_50x.fastq.gz -2 YT11_R2_001_trimq25_50x.fastq.gz --trusted-contigs Cminus_contigs.fasta --phred-offset 33 -k 21,33,55,77 -o Cminus_SPADES_Nanopore

#### 5. Evaluate the genome based on N50 and conserved orthologs with gVolante^6,7^

Tools: https://gvolante.riken.jp/analysis.html

#### 6. Run Redundans^8^ on the SPAdes assembly to selectively remove alternative heterozygous contigs and short contigs

python2 redundans.py -f scaffolds.fasta -o N5_spades_ONT_assembly_redundans --minLength 1000 --noscaffolding --nogapclosing

#### 7. Evaluate the new assembly after redundans with gVolante^6,7^

- Tools: https://gvolante.riken.jp/analysis.html
- Aassembly stats: https://gvolante.riken.jp/script/result.cgi?202106260431-6AN91SEAPYU7SWPF

#### 8. Scaffolding

##### a. Generate artificial matepairs* from 10X Genomics assembly (step 2) >10kb contigs

* Ryan, J. (2015b). Matemaker. Available online at: https://github.com/josephryan/ matemaker (accessed February 26, 2020).

###### Libraries with small insert size

matemaker --assembly=10kb_contigs_10x_canu.fa --insertsize=200 --out=10x_genomics_10kb.200

matemaker --assembly=10kb_contigs_10x_canu.fa --insertsize=500 --out=10x_genomics_10kb.500

matemaker --assembly=10kb_contigs_10x_canu.fa --insertsize=700 --out=10x_genomics_10kb.700

matemaker --assembly=10kb_contigs_10x_canu.fa --insertsize=1000 --out=10x_genomics_10kb.1k

matemaker --assembly=10kb_contigs_10x_canu.fa --insertsize=1200 --out=10x_genomics_10kb.1200

matemaker --assembly=10kb_contigs_10x_canu.fa --insertsize=1500 --out=10x_genomics_10kb.1500

matemaker --assembly=10kb_contigs_10x_canu.fa --insertsize=1700 --out=10x_genomics_10kb.1700

matemaker --assembly=10kb_contigs_10x_canu.fa --insertsize=2000 --out=10x_genomics_10kb.2k

matemaker --assembly=10kb_contigs_10x_canu.fa --insertsize=5000 --out=10x_genomics_10kb.5k

matemaker --assembly=10kb_contigs_10x_canu.fa --insertsize=10000 --out=10x_genomics_10kb.10k

matemaker --assembly=10kb_contigs_10x_canu.fa --insertsize=15000 --out=10x_genomics_10kb.15k

###### Libraries with large insert size

matemaker --assembly=10kb_contigs_10x_canu.fa --insertsize=2000 --out=10x_genomics_10kb.2k

matemaker --assembly=10kb_contigs_10x_canu.fa --insertsize=5000 --out=10x_genomics_10kb.5k

matemaker --assembly=10kb_contigs_10x_canu.fa --insertsize=7000 --out=10x_genomics_10kb.7k

matemaker --assembly=10kb_contigs_10x_canu.fa --insertsize=10000 --out=10x_genomics_10kb.10k

matemaker --assembly=10kb_contigs_10x_canu.fa --insertsize=12000 --out=10x_genomics_10kb.12K

matemaker --assembly=10kb_contigs_10x_canu.fa --insertsize=15000 --out=10x_genomics_10kb.15k

matemaker --assembly=10kb_contigs_10x_canu.fa --insertsize=17000 --out=10x_genomics_10kb.17k

matemaker --assembly=10kb_contigs_10x_canu.fa --insertsize=20000 --out=10x_genomics_10kb.20k

matemaker --assembly=10kb_contigs_10x_canu.fa --insertsize=22000 --out=10x_genomics_10kb.22k

matemaker --assembly=10kb_contigs_10x_canu.fa --insertsize=25000 --out=10x_genomics_10kb.25k

matemaker --assembly=10kb_contigs_10x_canu.fa --insertsize=27000 --out=10x_genomics_10kb.27k

matemaker --assembly=10kb_contigs_10x_canu.fa --insertsize=30000 --out=10x_genomics_10kb.30k

matemaker --assembly=10kb_contigs_10x_canu.fa --insertsize=32000 --out=10x_genomics_10kb.32k

matemaker --assembly=10kb_contigs_10x_canu.fa --insertsize=35000 --out=10x_genomics_10kb.35k

matemaker --assembly=10kb_contigs_10x_canu.fa --insertsize=37000 --out=10x_genomics_10kb.37k

matemaker --assembly=10kb_contigs_10x_canu.fa --insertsize=40000 --out=10x_genomics_10kb.40k

matemaker --assembly=10kb_contigs_10x_canu.fa --insertsize=42000 --out=10x_genomics_10kb.42k

matemaker --assembly=10kb_contigs_10x_canu.fa --insertsize=45000 --out=10x_genomics_10kb.45k

matemaker --assembly=10kb_contigs_10x_canu.fa --insertsize=47000 --out=10x_genomics_10kb.47k

matemaker --assembly=10kb_contigs_10x_canu.fa --insertsize=50000 --out=10x_genomics_10kb.50k

##### b. Create a libraries.txt file that can be used by SSPACE^9^ to scaffold the best assembly with the artificial matepairs:

###### Libraries with small insert size

lib1 10x_genomics_10kb.200.A.fq 10x_genomics_10kb.200.B.fq 200 0.25 FR

lib2 10x_genomics_10kb.500.A.fq 10x_genomics_10kb.500.B.fq 500 0.25 FR

lib3 10x_genomics_10kb.700.A.fq 10x_genomics_10kb.700.B.fq 700 0.25 FR

lib4 10x_genomics_10kb.1k.A.fq 10x_genomics_10kb.1k.B.fq 1000 0.25 FR

lib5 10x_genomics_10kb.1200.A.fq 10x_genomics_10kb.1200.B.fq 1200 0.25 FR

lib6 10x_genomics_10kb.1500.A.fq 10x_genomics_10kb.1500.B.fq 1500 0.25 FR

lib7 10x_genomics_10kb.1700.A.fq 10x_genomics_10kb.1700.B.fq 1700 0.25 FR

lib8 10x_genomics_10kb.2k.A.fq 10x_genomics_10kb.2k.B.fq 2000 0.25 FR

lib9 10x_genomics_10kb.5k.A.fq 10x_genomics_10kb.5k.B.fq 5000 0.25 FR

lib10 10x_genomics_10kb.10k.A.fq 10x_genomics_10kb.10k.B.fq 10000 0.25 FR

###### Libraries with large insert size

lib1 10x_genomics_10kb.2k.A.fq 10x_genomics_10kb.2k.B.fq 2000 0.25 FR

lib2 10x_genomics_10kb.5k.A.fq 10x_genomics_10kb.5k.B.fq 5000 0.25 FR

lib3 10x_genomics_10kb.7k.A.fq 10x_genomics_10kb.7k.B.fq 7000 0.25 FR

lib4 10x_genomics_10kb.10k.A.fq 10x_genomics_10kb.10k.B.fq 10000 0.25 FR

lib5 10x_genomics_10kb.12K.A.fq 10x_genomics_10kb.12K.B.fq 12000 0.25 FR

lib6 10x_genomics_10kb.15k.A.fq 10x_genomics_10kb.15k.B.fq 15000 0.25 FR

lib7 10x_genomics_10kb.17k.A.fq 10x_genomics_10kb.17k.B.fq 17000 0.25 FR

lib8 10x_genomics_10kb.20k.A.fq 10x_genomics_10kb.20k.B.fq 20000 0.25 FR

lib9 10x_genomics_10kb.22k.A.fq 10x_genomics_10kb.22k.B.fq 22000 0.25 FR

lib10 10x_genomics_10kb.25k.A.fq 10x_genomics_10kb.25k.B.fq 25000 0.25 FR

lib11 10x_genomics_10kb.27k.A.fq 10x_genomics_10kb.27k.B.fq 27000 0.25 FR

lib12 10x_genomics_10kb.30k.A.fq 10x_genomics_10kb.30k.B.fq 30000 0.25 FR

lib13 10x_genomics_10kb.32k.A.fq 10x_genomics_10kb.32k.B.fq 32000 0.25 FR

lib14 10x_genomics_10kb.35k.A.fq 10x_genomics_10kb.35k.B.fq 35000 0.25 FR

lib15 10x_genomics_10kb.37k.A.fq 10x_genomics_10kb.37k.B.fq 37000 0.25 FR

lib16 10x_genomics_10kb.40k.A.fq 10x_genomics_10kb.40k.B.fq 40000 0.25 FR

lib17 10x_genomics_10kb.42k.A.fq 10x_genomics_10kb.42k.B.fq 42000 0.25 FR

lib18 10x_genomics_10kb.45k.A.fq 10x_genomics_10kb.45k.B.fq 45000 0.25 FR

lib19 10x_genomics_10kb.47k.A.fq 10x_genomics_10kb.47k.B.fq 47000 0.25 FR

lib20 10x_genomics_10kb.50k.A.fq 10x_genomics_10kb.50k.B.fq 50000 0.25 FR

#### 9. Use SSPACE to scaffold the SPADes assembly with small insert size libraries

SSPACE_Basic_v2.0.pl -l libraries.txt -s N5_canu1_spades_lessthan1000bp_scaffolds.reduced_contaminated_removed.fasta -T 20 -k 5 -x 0 -a 0.7 -b N5_canu1_spades_lessthan1000bp_scaffolds_decontaminated_10x_sspace

#### 10. Use SSPACE to scaffold the SPADes assembly with large insert size libraries

SSPACE_Basic_v2.0.pl -l libraries.txt -s N5_canu1_spades_lessthan1000bp_scaffolds_decontaminated_10x_sspace.final.scaffolds.fasta -T 20 -k 5 -x 0 -a 0.7 -b N5_canu1_spades_lessthan1000bp_scaffolds_decontaminated_10x_sspace

#### 11. Break gaps > 10kb

perl /Users/teklelab/Documents/Softwares/JFR-PerlModules/scripts/break_big_gaps.pl N5_canu1_spades_lessthan1000bp_scaffolds_decontaminated_10x_sspace.final.scaffolds.fasta 10000 > N5_canu1_spades_lessthan1000bp_scaffolds_decontaminated_10x_sspace.final.scaffolds_gapbreak.fasta

#### 12. Rename genome and sort scaffolds

## Sort by scaffold length

remove_short_and_sort N5_canu1_spades_lessthan1000bp_scaffolds_decontaminated_10x_sspace.final.scaffolds_gapbreak.fasta 200 > N5_canu1_spades_lessthan1000bp_scaffolds_decontaminated_10x_sspace_scaffolds_gapbreak_sorted.fasta

## Replace definition lines

grep -c '^>' N5_canu1_spades_lessthan1000bp_scaffolds_decontaminated_10x_sspace_scaffolds_gapbreak_sorted.fasta | perl -ne '$num = scalar(split/|/); print "$num\n";'

## replace the deflines using the pad value

/Users/teklelab/Documents/Softwares/JFR-PerlModules/scripts/replace_deflines.pl --fasta=N5_canu1_spades_lessthan1000bp_scaffolds_decontaminated_10x_sspace_scaffolds_gapbreak_sorted.fasta --prefix=Cpenta_genomic_scaffolds --pad=5 > Cpen_scaffolds.v1.fa

#### 13. Run BLASTn^10^ (draft scaffods vs. nt database) to remove contaminated scaffolds

##### a. Download nt database (this database is large and may take a while)

perl update_blastdb.pl --decompress nt

##### b. Run BLASTn

export BLASTDB='/Users/teklelab/Desktop/N5_Genome_nt_blast_for_contaminants/nt' ## export path to the nt database

blastn -query N5_canu1_spades_lessthan1000bp_scaffolds_decontaminated_10x_sspace.final.scaffolds_gapbreak.fasta \

-db nt \

-evalue 1e-15 -outfmt "7 qseqid sseqid staxids sscinames scomnames qcovs evalue pident length sstart send sacc" \

-max_target_seqs 1 \

-out N5_canu1_spades_lessthan1000bp_scaffolds.reduced_blastn_nt.txt -num_threads 20

##### c. Manually remove any scaffolds that have significant Blast hits (>90% identity and >90% query coverage) to Bact, Virus, Archaea (see methods in main manuscript).

# PART 2: Genome Annotation

General Pipeline

1. Repeat mask the genome (as recommended in **BRAKER2^11-23^** use guide)

2. Align RNASeq to the genome to create a bam file 3.Also have proteins from closer species (Acanthamoeba) –> create a hint protein file 5.Run BRAKER on RepeatMasked genome.

#### 1. Align RNASeq to the genome to create a bam file using STAR^24^ aligner

##### a. Create index file

## create index file

STAR --runThreadN 15 \

--runMode genomeGenerate \

--genomeDir ./genome-index \

--genomeFastaFiles Cpen_scaffolds.v1.fa

##### b. Map RNA-seq reads to indexed assembly

STAR --runThreadN 15 \

--genomeDir ./00-genome-index \

--readFilesIn N5_R1_concat_alltranscriptomes_q28_trimmed40.fastq.gz,N5_R2_concat_alltranscriptomes_q28_trimmed40.fastq.gz \

--readFilesCommand gunzip -c

##### c. Conver sam to bam and sort alginment file

samtools view -u mapped.sam | samtools sort -o Cpen_RNA_seq_alignment_sorted_v2.bam

#### 2. Create a hint protein file using proteins from *A. castellanii*

ocean/projects/tra180030p/ytekle/Software/ProtHint/bin/prothint.py \

Cpen_scaffolds.v2.fasta \

GCF_000313135.1_Acastellanii.strNEFF_v1_protein.fa \

--workdir Acas_prothint_v2

#### 3. Run genome annotation using BRAKER2^11-23^

braker.pl --genome=Cpen_scaffolds.v2.fasta \

--bam=Cpen_RNA_seq_alignment_sorted_v2.bam \

--hints=./Acas_prothint_v2/prothint_augustus.gff --gff3 \

--etpmode \

--BAMTOOLS_PATH=/ocean/projects/tra180030p/ytekle/Software/bamtools/bin \

--cores=8

#### 4. Run BLASTp (C. minus proteins vs. nr database) to decontaminate (see *Part-2* 13-c)

##### a. Download nr database

perl update_blastdb.pl --decompress nr

##### b. Run BLASTp

export BLASTDB='/Volumes/Backup_Plus/N5_genome_project/Cminus_spades_ONT_reads_hybrid/nr' ##export path to nr database

blastp -query augustus.hints.aa -db nr -evalue 0.001 -outfmt "6 qseqid sseqid staxids sskingdoms sscinames covs evalue pident length sstart send sacc" -out Cpen_blastp_prot_1.txt -num_threads 20

##### c. Manually remove scaffolds containing significant sequences hit to bacteria, virus, and archaea

#### 5. Check the completeness with BUSCO^25^

Use tool: https://gvolante.riken.jp/analysis.html

**6. Functional classification predicted gene models and domain search**

EggNOG-mapper^26,27^ as implemented in OmicsBox v.2.0.29*. was used to classified likely homologs in and associated Clusters of Orthologous Groups (COGs) of our predicted gene models.

Domains of selected gene models were predicted using Hmmer web server v. 2.41.1^28^ against the reference proteome database with default parameters (https://www.ebi.ac.uk/Tools/hmmer/).

*OmicsBox - Bioinformatics made easy. BioBam Bioinformatics (Version 2.0.29). March 3, 2019. www. biobam.com/omicsbox

1 Weisenfeld, N. I., Kumar, V., Shah, P., Church, D. M. & Jaffe, D. B. Direct determination of diploid genome sequences. *Genome Res* **27**, 757-767, doi:10.1101/gr.214874.116 (2017).

2 Koren, S. *et al.* Canu: scalable and accurate long-read assembly via adaptive k-mer weighting and repeat separation. *Genome Res* **27**, 722-736, doi:10.1101/gr.215087.116 (2017).

3 Koren, S. *et al.* De novo assembly of haplotype-resolved genomes with trio binning. *Nat Biotechnol*, doi:10.1038/nbt.4277 (2018).

4 Nurk, S. *et al.* HiCanu: accurate assembly of segmental duplications, satellites, and allelic variants from high-fidelity long reads. *Genome Res* **30**, 1291-1305, doi:10.1101/gr.263566.120 (2020).

5 Bankevich, A. *et al.* SPAdes: a new genome assembly algorithm and its applications to single-cell sequencing. *J Comput Biol* **19**, 455-477, doi:10.1089/cmb.2012.0021 (2012).

6 Nishimura, O., Hara, Y. & Kuraku, S. gVolante for standardizing completeness assessment of genome and transcriptome assemblies. *Bioinformatics* **33**, 3635-3637, doi:10.1093/bioinformatics/btx445 (2017).

7 Nishimura, O., Hara, Y. & Kuraku, S. Evaluating Genome Assemblies and Gene Models Using gVolante. *Methods Mol Biol* **1962**, 247-256, doi:10.1007/978-1-4939-9173-0_15 (2019).

8 Pryszcz, L. P. & Gabaldon, T. Redundans: an assembly pipeline for highly heterozygous genomes. *Nucleic Acids Res* **44**, e113, doi:10.1093/nar/gkw294 (2016).

9 Boetzer, M. & Pirovano, W. SSPACE-LongRead: scaffolding bacterial draft genomes using long read sequence information. *BMC Bioinformatics* **15**, 211, doi:10.1186/1471-2105-15-211 (2014).

10 Altschul, S. F., W. Fish, W. Miller, E.W. Myers, and D.J. Lipman. Basic local alignment search tool. *J. Mol. Biol.* **215**, 403-410 (1990).

11 Hoff, K. J., Lange, S., Lomsadze, A., Borodovsky, M. & Stanke, M. BRAKER1: Unsupervised RNA-Seq-Based Genome Annotation with GeneMark-ET and AUGUSTUS. *Bioinformatics* **32**, 767-769, doi:10.1093/bioinformatics/btv661 (2016).

12 Bruna, T., Hoff, K. J., Lomsadze, A., Stanke, M. & Borodovsky, M. BRAKER2: automatic eukaryotic genome annotation with GeneMark-EP+ and AUGUSTUS supported by a protein database. *NAR Genom Bioinform* **3**, lqaa108, doi:10.1093/nargab/lqaa108 (2021).

13 Hoff, K. J., Lomsadze, A., Borodovsky, M. & Stanke, M. Whole-Genome Annotation with BRAKER. *Methods Mol Biol* **1962**, 65-95, doi:10.1007/978-1-4939-9173-0_5 (2019).

14 Stanke, M., Diekhans, M., Baertsch, R. & Haussler, D. Using native and syntenically mapped cDNA alignments to improve de novo gene finding. *Bioinformatics* **24**, 637-644, doi:10.1093/bioinformatics/btn013 (2008).

15 Stanke, M., Schoffmann, O., Morgenstern, B. & Waack, S. Gene prediction in eukaryotes with a generalized hidden Markov model that uses hints from external sources. *BMC Bioinformatics* **7**, 62, doi:10.1186/1471-2105-7-62 (2006).

16 Buchfink, B., Xie, C. & Huson, D. H. Fast and sensitive protein alignment using DIAMOND. *Nat Methods* **12**, 59-60, doi:10.1038/nmeth.3176 (2015).

17 Li, H. *et al.* The Sequence Alignment/Map format and SAMtools. *Bioinformatics* **25**, 2078-2079, doi:10.1093/bioinformatics/btp352 (2009).

18 Barnett, D. W., Garrison, E. K., Quinlan, A. R., Stromberg, M. P. & Marth, G. T. BamTools: a C++ API and toolkit for analyzing and managing BAM files. *Bioinformatics* **27**, 1691-1692, doi:10.1093/bioinformatics/btr174 (2011).

19 Lomsadze, A., Burns, P. D. & Borodovsky, M. Integration of mapped RNA-Seq reads into automatic training of eukaryotic gene finding algorithm. *Nucleic Acids Res* **42**, e119, doi:10.1093/nar/gku557 (2014).

20 Bruna, T., Lomsadze, A. & Borodovsky, M. GeneMark-EP+: eukaryotic gene prediction with self-training in the space of genes and proteins. *NAR Genom Bioinform* **2**, lqaa026, doi:10.1093/nargab/lqaa026 (2020).

21 Lomsadze, A., Ter-Hovhannisyan, V., Chernoff, Y. O. & Borodovsky, M. Gene identification in novel eukaryotic genomes by self-training algorithm. *Nucleic Acids Res* **33**, 6494-6506, doi:10.1093/nar/gki937 (2005).

22 Gotoh, O. A space-efficient and accurate method for mapping and aligning cDNA sequences onto genomic sequence. *Nucleic Acids Res* **36**, 2630-2638, doi:10.1093/nar/gkn105 (2008).

23 Iwata, H. & Gotoh, O. Benchmarking spliced alignment programs including Spaln2, an extended version of Spaln that incorporates additional species-specific features. *Nucleic Acids Res* **40**, e161, doi:10.1093/nar/gks708 (2012).

24 Dobin, A. *et al.* STAR: ultrafast universal RNA-seq aligner. *Bioinformatics* **29**, 15-21, doi:10.1093/bioinformatics/bts635 (2013).

25 Simao, F. A., Waterhouse, R. M., Ioannidis, P., Kriventseva, E. V. & Zdobnov, E. M. BUSCO: assessing genome assembly and annotation completeness with single-copy orthologs. *Bioinformatics* **31**, 3210-3212, doi:10.1093/bioinformatics/btv351 (2015).

26 Cantalapiedra, C. P., Hernandez-Plaza, A., Letunic, I., Bork, P. & Huerta-Cepas, J. eggNOG-mapper v2: Functional Annotation, Orthology Assignments, and Domain Prediction at the Metagenomic Scale. *Mol Biol Evol* **38**, 5825-5829, doi:10.1093/molbev/msab293 (2021).

27 Huerta-Cepas, J. *et al.* Fast Genome-Wide Functional Annotation through Orthology Assignment by eggNOG-Mapper. *Mol Biol Evol* **34**, 2115-2122, doi:10.1093/molbev/msx148 (2017).

28 Potter, S. C. *et al.* HMMER web server: 2018 update. *Nucleic Acids Res* **46**, W200-W204, doi:10.1093/nar/gky448 (2018).
